# Editorial for the Special Issue “Current Research on Cancer Biology and Therapeutics: 2nd Edition”

**DOI:** 10.3390/ijms252111777

**Published:** 2024-11-02

**Authors:** Rafael Coveñas

**Affiliations:** Laboratory of Neuroanatomy of the Peptidergic Systems, Institute of Neurosciences of Castilla and León (INCYL), University of Salamanca, c/Pintor Fernando Gallego 1, 37007 Salamanca, Spain; covenas@usal.es; Tel.: +34-923294400 (ext. 1856)

In the second edition of this Special Issue, several promising antitumor strategies have been presented in addition to those reported in the first edition, in which several compounds (acetylcorynoline, BaP1, sarco/endoplasmic reticulum calcium ATPase inhibitors, neuropeptide Y, neuropeptide Y antagonists, neurokinin-1 receptor antagonists) exerting antitumor effects against colorectal cancer, papillary thyroid carcinoma, cholangiocarcinoma, Ewing sarcoma, liver cancer, and breast cancer were reported [1]. In the second edition, the participating researchers have focused their studies on different therapeutic approaches to fighting papillary thyroid carcinoma [2], colorectal cancer [3,4], breast cancer [5,6], prostate cancer [5], pancreatic cancer [6], glioma [7], bladder cancer [5], and ovarian cancer [8]. Another of the studies has suggested a way to enhance the effectiveness of anticancer compounds by inducing multipolar mitotic spindles to fight cancer [9]. Together, the first and second editions show a plethora of promising lines of antitumor research to improve both the diagnosis and treatment of several types of cancer.

Peter Lakatos and his group (Semmelweis University, Budapest, Hungary) have studied the fusion mutations that occur in papillary thyroid carcinoma, the predominant subtype of thyroid cancer which also is the most frequent endocrine malignancy [2]. They stated that molecular therapies can be used as effective strategies to adequately fight tumors when targetable gene mutations, originating from gene fusions, occur. After applying various molecular diagnostics techniques such as next-generation sequencing, gene fusions (*RET*, *NTRK3*, *CCDC6*, *ETV6*, *MET*, *ALK*, *NCOA4*, *EML4*, *SQSTM1*) were detected in 27% of papillary thyroid carcinoma tissue samples. These genes were associated with a history of Hashimoto’s disease, smaller tumor size, radioiodine therapy, thyroidectomy type, obstructive pulmonary disease, multifocality, goiter history, microcarcinoma character, sex, endometriosis, hypothyroidism history, and a medical history of thyroid/parathyroid adenoma [2]. The study highlights the relationships between clinicopathological features and fusion mutations and the importance of incorporating molecular profiling into the routine management of papillary thyroid carcinoma because the early detection of these mutations would help practitioners to make adequate surgical decisions and choose appropriate therapeutic strategies to follow, improving the clinical outcomes of papillary thyroid carcinoma patients.

Tumors often develop resistance to chemotherapy drugs such as cisplatin; hence, new anticancer strategies or synergistic combinations with other compounds are needed [3]. The combination of several compounds is essential to develop future potential antitumor strategies, and this has been carried out by the group led by Igor Kovalchuk (University of Lethbridge, Lethbridge, Canada). This group has studied the synergistic antitumor action of a combination of cisplatin and cannabinoid delta-9-tetrahydrocannabinol (THC), as well as extract #18 (a high-THC *Cannabis sativa* extract), against human colorectal cancer cell lines (LS-174T, HCT-116, HT-29). This extract blocked cell growth and its effect was higher when compared with that observed after the administration of THC alone. The study also showed the effect of an antagonistic interaction between the high-THC cannabis extract and cisplatin on cancer cells and the authors suggested that this could be associated with gene transcription alterations related to the tumor pathways involved in drug resistance, DNA repair (Rad52), and cell death (Bcl-2, caspase 10, Bad). The study concluded that the combination of extract #18/cisplatin counteracted the antitumor effects mediated by the chemotherapy drug or the extract #18 alone on tumor cells, because such a combination promoted prosurvival mechanisms [3]. This study reveals the importance of unmasking the molecular processes promoted after the co-administration of several compounds and, in addition, it will serve to increase knowledge on the specific mechanisms involved in drug interactions. Maximino Redondo and his group (University of Málaga, Málaga, Spain) have reviewed the identification of biomarkers in colorectal cancer through proteomics [4]. Due to the molecular complexity of this disease, this group highlights that, in colorectal cancer, proteomics could be a very useful tool for early diagnosis, to increase knowledge of the molecular alterations involved, and for a better differentiation of the different colorectal cancer subtypes. Collectively, this will allow us to develop personalized antitumor treatments, to provide crucial information for clinical decisions and treatments, and to suggest new anticancer strategies. This review points out that targeted therapies combined with immune checkpoint inhibitors/standard chemotherapy are promising treatments against colorectal cancer but that their effectiveness is associated with the molecular characteristics of each patient suffering from the disease [4]. In this sense, protein biomarkers (STK4, NCR1, CD147, ERGIC1, ACTBL2, DPEP1) located in tissue, urine, plasma, or serum will play a crucial role in the early detection and treatment of colorectal cancer.

The Ana Salomé Pires group (University of Coimbra, Coimbra, Portugal) has studied the migration, viability, cell proliferation, and death profile of human bladder (TCCSUP, HT1376), prostate (PC3, LNCaP), and breast (MCF7) cancer cell lines after the administration of local anesthetics (levobupivacaine, ropivacaine, lidocaine) [5]. These anesthetics exerted anticancer effects against the previously mentioned cell lines and an antitumor synergistic action was observed against metastatic prostate cancer cells when docetaxel and local anesthetics were co-administered. This combined therapy reduced cell viability, augmented cell death by necrosis, and blocked the migration of prostate cells. Accordingly, anesthetics increased the antitumor efficacy of docetaxel, and this suggests that local anesthetics could reduce the toxicity and side-effects of this chemotherapy drug [5]. This research group also reported the clinical outcomes of opioid use in patients with metastatic prostate cancer. The chronic administration of opioids was related to reduced overall survival and progression-free survival [5]. Although more studies are needed to understand the clinical impact of opioid analgesics on prostate cancer patients, the study suggests the potential use of local anesthetics as an antitumor co-adjuvant treatment.

The Vilma Petrikaité group (Vilnius University, Vilnius, Lithuania) has shown the actions exerted by 4-(dimethylamino) phenyl-5-oxopyrrolidines on colony formation, cell proliferation and migration, and tumor spheroid growth in human triple-negative breast (MDA-MB-231) and ductal pancreatic carcinoma (Panc-1) cancer cells [6]. Thus, a series of 4-((dimethylamino) phenyl) pyrrolidin-2-ones bearing hydrazide, hydrazone, and heterocyclic moieties were synthesized and their antitumor effects were studied. These compounds reduced the viability of both cancer cells, completely disturbed cell colony growth (MDA-MB-231), which was a particularly strong effect in the case of Panc-1 cells, blocked spheroid growth (Panc-1), and reduced spheroid cell viability (MDA-MB-231) [6]. However, none of these compounds affected cancer cell migration. This study opens the door to performing more in-depth studies to better understand the activity–structure relationships between the compounds tested and the mechanisms of action involved, and confirms the importance of synthesizing new compounds showing a high antitumor activity.

Sánchez and co-workers (University of Salamanca, Salamanca, Spain) have reviewed the involvement of peptides in glioma development [7]. Oncogenic peptides (adrenomedullin, angiotensin, neurotensin, substance P) promote cancer cell proliferation, migration, and angiogenesis, whereas anticancer peptides (methionine-enkephalin, somatostatin, corticotropin-releasing factor) exert the opposite effects. Other peptides (oxytocin, vasoactive intestinal peptide) have a dual action, exerting proliferative and antiproliferative effects on glioma cells. Moreover, glioma cells overexpress peptide receptors which are a useful tool for the diagnosis, research, management, and treatment of patients with a glioma [7]. This review highlights the important roles that peptidergic systems play in glioma development because these systems are diagnostic markers and potential therapeutic targets for treating gliomas using peptide receptor antagonists/peptides or peptide receptor agonists. In addition, the repurposing of certain antihypertensive or anti-emetic drugs and the use of peptide receptor antagonists alone or in combination with chemotherapy/radiotherapy are also suggested for the treatment of glioma.

Simona Frezzini and co-workers (Veneto Institute of Oncology, Padova, Italy) have studied the action of poly-ADP ribose polymerase (PARP) inhibitors in brain metastases from epithelial ovarian cancer, the deadliest gynecological malignancy, through six case reports [8]. This research group studied cases showing both BRCA wild-type and BRCA-mutated epithelial ovarian cancer spreading to the central nervous system with single or multiple lesions and most of them showing intracranial-only disease. In all cases studied, the patients experienced clinical benefits after receiving PARP inhibitor maintenance for a long period of time within a multimodal treatment approach [8]. The authors discuss the management of the disease, future therapeutic challenges, and personalized treatment plans, and highlight the importance of an in-depth understanding of the role that PARP inhibitors play in the disease according to the findings reported in the six cases studied [8].

The Kai-Ping Chang group (Chang Gung University, Taoyuan, Taiwan) proposed the enhancement of the effectiveness of anticancer compounds promoting multipolar mitosis spindles by suppressing anaphase-promoting complex/cyclosome-cell division cycle 20 (APC/C-CDC20) activity [9]. This suppression increases the sensitivity to kinesin family 18a inhibition, which suppresses the multipolar mitotic spindles in tumor cells [9]. The authors suggest the blockade of APC/C-CDC20 activity to delay, but not inhibit, anaphase entry in mitosis. This delay can increase multipolar spindle-induced cell death in genomically unstable cells, whereas in stable healthy cells, delayed anaphase entry may inhibit the level of multipolar spindles promoted by anticancer compounds and lower mitotic cytotoxicity. Accordingly, the authors state that this could be tested by using anticancer drugs (vinorelbine, eribulin, paclitaxel) or kinesin family 18a inhibitors (sovilnesib) [9]. The authors also suggest that previous compounds could be tested in combination with APC/C-CDC20 inhibitors (CP5V, apcin, proTAME, peptide inhibitors) in cancer cell line models. This is a future line of research that deserves to be fully investigated.

The results presented by the researchers participating in the second edition of this Special Issue increase our knowledge about several mechanisms involved in cancer and open the door to developing and exploring new lines of antitumor research that could be transferred to clinical practice in the future. In this Special Issue, the integration of molecular therapies into the routine management of tumors, the importance of unmasking the molecular mechanisms promoted when several compounds are co-administered, the identification of biomarkers through proteomics, the importance of knowing the specific mechanisms involved in drug interactions, the use of local anesthetics, the antitumor effects of 4-(dimethylamino) phenyl-5-oxopyrrolidine derivatives, and the importance of oncogenic and anticancer peptides as well as peptide receptor agonists/antagonists, PARP inhibitors, and the control of APC/C-CDC20 activity are proposed as therapeutic strategies to fight papillary thyroid carcinoma, colorectal cancer, bladder cancer, prostate cancer, breast cancer, pancreatic cancer, glioma, and ovarian cancer. These strategies will allow for early detection and diagnosis, better knowledge of the molecular alterations involved, better differentiation of the different cancer subtypes, developing personalized antitumor treatments, providing crucial information for clinical/surgical decisions and treatments, and for developing potential antitumor options for translational research.
